# Adverse Health Effects of the Long-Term Simultaneous Exposure to Arsenic and Particulate Matter in a Murine Model

**DOI:** 10.1155/2024/5391316

**Published:** 2024-05-09

**Authors:** Cesar Rivas-Santiago, Maria Gallegos-Bañuelos, Irving Trejo-Ramos, Nancy Solís-Torres, Raúl Quintana-Belmares, Noé Macías-Segura, Héctor Gutiérrez-Bañuelos, Lorena Troncoso-Vazquez, Bruno Rivas-Santiago, Irma Gonzalez-Curiel

**Affiliations:** ^1^CONAHCYT-Academic Unit of Chemical Sciences, Autonomous University of Zacatecas, Zacatecas 98085, Mexico; ^2^Sciences and Chemical Technology, Chemistry Sciences School, Autonomous University of Zacatecas, Zacatecas 98085, Mexico; ^3^Pharmacobiology, Chemistry Sciences School, Autonomous University of San Luis Potosi, San Luis Potosi 78210, Mexico; ^4^Basic Research, National Institute of Cancerology, Mexico City 14080, Mexico; ^5^Service and Department of Immunology, Faculty of Medicine and University Hospital, Autonomous University of Nuevo León, Nuevo León, 66450, Mexico; ^6^Veterinary Medicine and Zootechnics School, Autonomous University of Zacatecas, Zacatecas 98085, Mexico; ^7^Histology Laboratory, Mexican Social Security Institute-IMSS, Zacatecas 98085, Mexico; ^8^Biomedical Research Unit-Zacatecas-IMSS, Mexican Social Security Institute, Zacatecas 98085, Mexico

## Abstract

PM_2.5_ and arsenic are two of the most hazardous substances for humans that coexist worldwide. Independently, they might cause multiple organ damage. However, the combined effect of PM_2.5_ and arsenic has not been studied. Here, we used an animal model of simultaneous exposure to arsenic and PM_2.5_. Adult Wistar rats were exposed to PM_2.5_, As, or PM_2.5_ + As and their corresponding control groups. After 7, 14, and 28 days of exposure, the animals were euthanized and serum, lungs, kidneys, and hearts were collected. Analysis performed showed high levels of lung inflammation in all experimental groups, with an additive effect in the coexposed group. Besides, we observed cartilaginous metaplasia in the hearts of all exposed animals. The levels of creatine kinase, CK-MB, and lactate dehydrogenase increased in experimental groups. Tissue alterations might be related to oxidative stress through increased GPx and NADPH oxidase activity. The findings of this study suggest that exposure to arsenic, PM_2.5_, or coexposure induces high levels of oxidative stress, which might be associated with lung inflammation and heart damage. These findings highlight the importance of reducing exposure to these pollutants to protect human health.

## 1. Introduction

Environmental pollution is a growing public health concern worldwide, encompassing greenhouse gas emissions, indoor and outdoor air pollution, waste management, and water pollution. Rapid global urbanization and industrialization have led to alarming levels of environmental pollutants that pose a significant risk to human health. Environmental pollution is estimated to contribute to one out of six deaths globally, equating to 9 million premature deaths each year [1]. In addition, exposure to environmental pollutants can cause perinatal, respiratory, cardiovascular, malignant, and mental disorders [2].

The World Health Organization (WHO) has identified ten chemical pollutants that pose significant public health concerns. Among these, arsenic (As) in water and air pollution are prevalent and dangerous to human health. Arsenic is a toxic metalloid widely present in soil and groundwater, affecting agriculture, food, and drinking water. Chronic exposure to arsenic has been linked to skin, liver, bladder, and lung cancer, cardiovascular diseases, cognitive deficiencies, and diabetes mellitus [3–7]. Arsenic exposure disrupts various metabolic pathways, with oxidative stress playing a central role in developing associated diseases [8–11]. However, in the natural environment, exposure to arsenic is not limited to a single pollutant but instead occurs concurrently with other pollutants that reach the body via the same or different routes.

Particulate matter (PM) is one of the most significant air pollutants, contributing to 4.2 million deaths annually worldwide. PM is a mixture of liquid and solid compounds released into the air by natural and anthropogenic sources, with the composition varying according to geography, season, and other factors [12, 13]. PM is classified based on size, PM_10_ (≤10 *μ*m), PM_2.5_ (≤2.5 *μ*m), and ultrafine particles or PM_0.1_ (≤0.1 *μ*m). All have different toxic effects on the body based on their capacity to reach deep into the respiratory system, and PM_2.5_ and PM_0.1_ can reach terminal bronchioles and even pass through to the circulatory system, with composition and size determining the health outcomes [14, 15].

While previous studies have demonstrated the harmful effects of individual pollutants, studying the effects of simultaneous exposure to various pollutants is crucial. In this study, we propose a murine model of concurrent exposure to PM (intratracheally) and inorganic arsenic (orally) to investigate morphological alterations in the lungs, heart, and kidneys, as well as oxidative stress markers induced by simultaneous exposure to these pollutants. Using differential gene expression analysis, we constructed a protein-protein interaction network from datasets of rats exposed to PM_2.5_ or arsenic selected from the Gene Expression Omnibus (GEO) repository observing similarities with our results.

## 2. Materials and Methods

### 2.1. Sampling of Air Pollution

PM_2.5_ was collected in 2016 at the Atmospheric Science Center (ASC) in Mexico City (CMX). The collection was performed using a continuously operating high-volume PM_2.5_ sampler (TE6070V-2.5. Tisch Environmental, Inc.; Village of Cleves, OH, USA) with an airflow rate of 1.13 m^3^/min. PM was collected on nitrocellulose membranes (Sartorius, Gottingen, Germany) with a nominal pore size of 3 *μ*m. The membranes were gently scraped using a surgical blade to collect the particles, which were placed in endotoxin-free glass vials after recovery. All PM_2.5_ samples collected were pooled and stored in a baked glass vial in the dark at 4°C until their use [16].

### 2.2. Chemical and Biological Analysis of PM Samples

The elemental composition of PM_2.5_ samples was determined by X-ray fluorescence (XRF) analysis using an Oxford Instrument X-ray tube with an Rh anode and an Amptek Si-PIN X-ray detector (resolution 160 eV at 5.9 keV). The obtained results were integrated with the software WINQXAS for XRF [17]. The polycyclic aromatic hydrocarbons' (PAHs) content was analyzed by high-resolution liquid chromatography in one milligram of the PM sample. Samples were extracted with 30 mL of dichloromethane in a microwave oven (CEM, model: MARS X) with a power of 1200 W, 100 psi of pressure, and a temperature of 115°C and the extract was analyzed using an HPLC, Agilent HP, 1100 series with an automatic sample injector and fluorescence detector and a Nucleosil column (Macherey–Nagel, 265 mm, 100-5 C18 PAH) [17]. Endotoxin levels were analyzed by the *Limulus amebocyte* lysate Kinetic-QCL method (Lonza Kinetic-QCL) in one milligram of the PM sample. In brief, the PM was suspended in 1 mL of 50 mM Tris buffer; then, the sample was sonicated for one hour at 22°C, vortexing 1 min every 15 min before running the quantitative method following the instructions provided by the supplier. The samples were analyzed in duplicate using sterile endotoxin-free 96-well microplates and *Escherichia coli* O55: B5 endotoxin as the standard (10 endotoxin units per mg).

### 2.3. Particulate Matter and Sodium Arsenite Preparation for *In Vivo* Administration

PM_2.5_ was weighed directly in a prewashed and baked (4 h at 200°C) glass flask using a microbalance (Pioneer Precision; OHAUS Corporation, NJ, USA). For animal exposure, the stock suspension of PM_2.5_ (640 *μ*g/mL) was prepared in a sterile saline solution. Before its use, the stock was sonicated for 30 min (BioSonic, model UC100; Whaledent/COLTENE, Cuyahoga, OH, USA).

We diluted 70 *μ*g of sodium arsenite (NaAsO_2_) in one liter of purified water for the murine model oral exposure.

### 2.4. Animal and Ethics Statement

All animal procedures were performed following the Autonomous University of Zacatecas ethical standards and conducted according to the guidelines of Care and Laboratory Animal Use and the Mexican Official Standard (NOM-062-ZOO-1999), after approval by the Ethics Committee of the Autonomous University of Zacatecas (HSC-UAZ) Health Sciences Research Area, registered with the protocol no. ACS/UAZ/086/2020. In addition, Animal Research: Reporting of *In Vivo* Experiments (ARRIVE) guidelines for the report of the results of animal experiments were followed [18].

### 2.5. The Animal Model for Simultaneous Exposure to Arsenic and Particulate Matter

Sixty adult Wistar rats weighing 440–460 grams were randomly assigned to five groups (12 rats each) and maintained in the animal facilities under controlled light (12 h light/dark cycle), temperature, and humidity. Commercial food (LabDiet 5001, St. Louis, MO, USA) and water were provided *ad libitum*. Each group created received the treatment as follows. The oral control (OC) group was exposed to purified drinking water. The intratracheal control (IC) was exposed to purified drinking water and saline solution intratracheally. The As group was only exposed to As in drinking water (70 *μ*g of NaAsO_2_/L) which was provided *ad libitum* on a daily basis. The PM group was only exposed intratracheally to PM_2.5_ (64 *μ*g/100 *μ*L) every other day. Finally, the As + PM_2.5_ group was exposed orally to As in drinking water and intratracheally to PM_2.5_ every other day. All animals subjected to intratracheal exposure, whether receiving saline solution or PM, were anesthetized by inhalation of 0.1 mL of sevoflurane (Savannlab®, PA, USA), and after anesthesia, a flexible tracheal cannula (model: Canula Alpha-Chymotrypsin TROUTMAN flexible 22GA, Inami Co., Tokyo, JPN), was used for the administration of their respective treatments.

After 7, 14, and 28 days of exposure to the conditions mentioned above, four rats per group were euthanized by exsanguination, and the blood collected was stored for further analysis. Then, kidneys, lungs, and hearts were collected and stored in 10% formalin for morphological analysis.

### 2.6. Preparation of Tissues for Histology/Morphology

Right or left lungs from three different animals per group were perfused intratracheally with ethyl alcohol (JT Baker, Avantor, Radnor, PEN, USA) and stored submerged in ethyl alcohol until their use. At the same time, the kidneys and the hearts were fixed and stored in 10% formalin, then embedded in paraffin (Paraplast PLU, Leica Biosystems, LD, GER) for 3 *μ*m thick histological tissue sections, using a manual rotatory microtome. As described elsewhere [19], tissue sections were stained with hematoxylin and eosin. Morphology and cellular infiltration were determined using an automatic image analyzer (Axiovert Vision Release 4.8.2 Software, Zeiss, GER).

### 2.7. Serum Biochemical Analyses

For the evaluation of kidney and heart function, biochemical parameters such as urea (REF, 4460715190), creatinine (REF, 4810716190), total protein (REF, 3183734190), albumin (REF, 3183688122), creatine kinase (CK, REF, 7190794190), creatine kinase-MB (CK-MB, REF, 7190808190), and lactate dehydrogenase (LDH, REF, 3004732122) were assessed in the serum of four rats from each condition. The biochemical parameters were determined using commercial kits following the instructions provided by the supplier (Roche Diagnosis, La Jolla, CA, USA). All measurements used a multichannel photometric unit Cobas 501 (Roche Diagnosis, Basel, Swiss).

### 2.8. Oxidative Stress Markers

The antioxidant activity of the glutathione peroxidase (Glutathione Peroxidase Assay Kit, AB102530, Abcam) and NADPH oxidase 4 (NOX4, ELISA kit, MBS1600298, MyBioSource) in the serum of experimental and control animals at each time point was assessed by a colorimetric method and sandwich ELISA, respectively, following instructions provided by the distributor. In brief, for colorimetric assay, the serum collected was added to a well containing colorimetric reaction mix (Assay buffer, 40 mM NADPH solution, GR solution, and GSH solution). Then, the plate was incubated for 15 minutes at room temperature, followed by adding a cumene hydroperoxide solution. Finally, spectrophotometric determinations were conducted at 340 nm every minute for five minutes.

For NOX4 sandwich ELISA, 96-well plates were coated with specific antibodies against NOX4 and then the serum samples were added and incubated. After that, a biotinylated NOX4 detector antibody was added to each well; after incubation, wells were washed three times, and the avidin-HRP conjugate was added. Following the wash cycle and incubation, TMB substrate was added, and after 10–20 minutes of incubation, a stop solution was added. Optical density absorbance was read at 450 nm with a wavelength correction set to 540 nm.

### 2.9. Dataset Selection from GEO Repository

Gene expression datasets were selected from the Gene Expression Omnibus (GEO) repository (https://www.ncbi.nlm.nih.gov/geo/). In addition, we included datasets with the following inclusion criteria: male rats exposed to arsenic (sodium (meta) arsenite, 1 ppm), male rats exposed to PM_2.5_, expression profiling by array, RNA sample from primary rat hepatocytes, and *Rattus norvegicus* (organism), containing at least three animals per group, including an experimental control group. In addition, we identified two datasets that fulfill the inclusion criteria: the GSE178513 dataset for the rat model of PM_2.5_ exposure and the GSE19662 dataset for arsenic exposure.

### 2.10. Differentially Expressed Gene (DEG) Analysis and Identification

The unsupervised gene expression analysis was performed using the online tool GEO2R (https://www.ncbi.nlm.nih.gov/geo/geo2r/). This platform is an interactive web tool for comparing two or more groups of gene expression samples in a GEO series based on the GEOquery and Limma R packages [20–22]. We compared exposed rats with As or PM_2.5_ and each group with their healthy internal control in the dataset. After downloading the gene expression analysis, thresholds of log2FC >1.0, <−1.0, and *P* value <0.05 were considered statistically significant.

### 2.11. Protein-Protein Interaction Network Construction

To further understand and enrich the analysis, the potential interaction between the statistically significant DEGs (upregulated and downregulated, separately) and the corresponding coexpressed genes is evaluated using the GeneMANIA platform (https://genemania.org/). First, we took the DEGs up or down, selected *Rattus norvegicus* as the organism, and ran the protein-protein interaction. Once we got the genes that interacted among the DEGs, we evaluated the gene ontology of all these genes to identify the biological function of the genes by using the PANTHER classification system (https://pantherdb.org/geneListAnalysis.do).

### 2.12. Statistical Analysis

A Shapiro–Wilk normality test was performed to determine the distribution in each group. A one-way ANOVA followed by a Sidak posttest was performed to compare % lung inflammation, cardiac and renal profile parameters, and GPx and NOX2 activities between different exposure times and treatments. Statistically significant differences were considered when the value of *p* < 0.05. The GraphPad statistical package, version 6.0 for Windows, was used.

## 3. Results

### 3.1. Chemical and Biological Composition of Urban PM_2.5_

The PM_2.5_ samples collected in 2016 at the Atmospheric Science Center (ASC) in Mexico City had high levels of aluminum (Al), calcium (Ca), copper (Cu), iron (Fe), lead (Pb), nickel (Ni), potassium (K), selenium (Se), silicon (Si), sulphur (S), vanadium (V), and elements and metals related to combustion and industrial activity (Table 1).

The PAH levels found in PM_2.5_ showed a high content of compounds from the incomplete combustion of organic matter (Table 2). Levels of endotoxins in this PM sample were 12.6 EU/mg.

### 3.2. Histological Alterations Caused by the Coexposure to Arsenic and Particulate Matter

**Figure 1:**
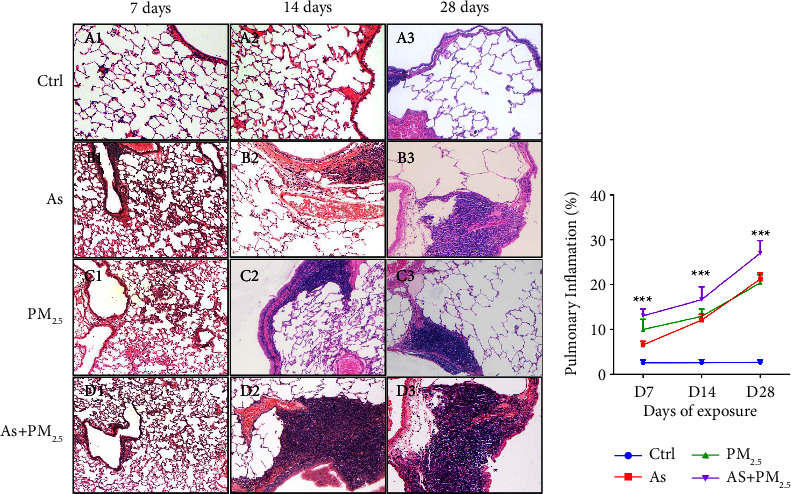
Effect of exposure to As and PM_2.5_ and the coexposure to As + PM_2.5_ in lung inflammation. The lung inflammation was analyzed by histological morphometric analysis. (a) The control group (A1 to A3) did not show lung inflammation areas at experimental time-points, whereas those exposed to As (B1 to B3), PM2.5, and coexposed to As and PM_2._5 (C1 to C3) showed a higher lung inflammation area which increases with the time of exposure. (b) The percentage area of total lung affected by inflammation was calculated at 7, 14, and 28 days in experimental and control groups, *n* = 4 per group. Data are presented as mean and SD. ^*∗∗∗*^*p* < 0.001 vs control.

Lung inflammation caused by exposure to As and PM_2.5_ and the coexposure to As and PM_2.5_ were assessed by histopathological analysis of lung sections at different points in the sections stained with hematoxylin-eosin. We observed higher cellular infiltration levels in the As + PM_2.5_ group, observing higher infiltration levels in a time-dependent manner (Figure 1) than the groups exposed to As or PM_2.5_ alone.

**Figure 2:**
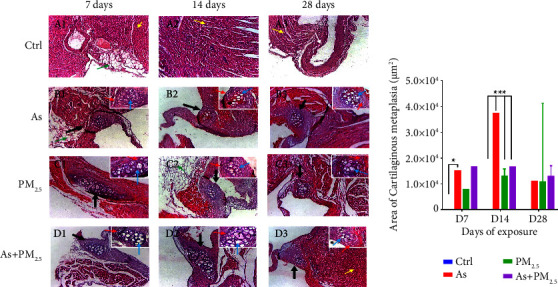
Histology of the heart from 28-days exposed rats to arsenic, PM_2.5_, and arsenic + PM_2.5_. Cross-section of hearts with hematoxylin-eosin staining and the hearts from control rats (A1–A3) did not show morphological alterations. The hearts from As (B1–B3), PM_2.5_ (C1–C3), and As + PM2.5 (D1–D3) exposed rats showed cartilaginous metaplasia areas (black arrow). The cartilaginous metaplasia areas in the hearts of As, PM_2.5_, and As + PM_2.5_ exposed rats were measured after 7, 14, and 28 days of exposure (b). *n* = 4 per group. Data are presented as median(range). ^*∗*^*p* < 0.05 and ^*∗∗∗*^*p* < 0.001 vs control.

In the histological analysis of heart tissue, we observed cartilaginous metaplasia only in exposed groups to As, PM_2.5_, and As + PM_2.5_, where fibrous connective tissue is replaced by hyaline cartilaginous tissue. In addition, we observed a hypertrophic state and irregular distribution of chondrocytes (Figure 2) in hearts from the same experimental groups. Interestingly, the area of cartilaginous metaplasia in the three exposed groups differs over time. Groups exposed to As and As + PM_2.5_ showed cartilaginous metaplasia that peaked at 14 days. However, PM_2.5_ exposed groups did not show significant increments in the cartilaginous metaplasia area (area of cartilaginous metaplasia, *μ*m^2^).

We did not find alterations compared with control groups after kidney histological analysis.

### 3.3. Biochemical Profiles

**Figure 3:**
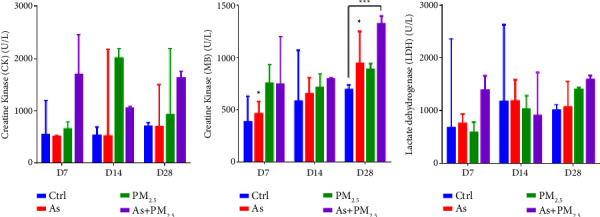
Biochemical test for cardiac function. The serum levels of CK (a), CK-MB (b), and LDH (c) were assessed after 7, 14, and 28 days in the rats exposed to As, PM_2.5_, or As + PM_2.5_ exposure *n* = 4 per group. Data are presented as median (range). ^*∗*^*p*=0.029, As (D7) *vs* As (D28). ^*∗∗∗*^*p*=0.0006 As + PM_2.5_ (D28) *vs* control (D28), respectively.

The cardiac function analysis was determined by quantifying creatine kinase (CK), creatine kinase-MB (CK-MB) isoenzyme, and lactate dehydrogenase (LDH) in the serum of control and exposed Wistar rats. The CK levels did not show significant changes in any experimental condition assessed (Figure 3(a)); nevertheless, levels of CK-MB in As + PM_2.5_ increased compared to other groups since day 7 of exposure (*p*=0.006). However, we observed a time-dependent increasing trend in CK-MB levels of serum from rats exposed to As, PM_2.5_, and As + PM_2.5_. The most significant change observed in all conditions was after 28 days of exposure. Interestingly, the group simultaneously exposed to PM_2.5_ and As has the highest rise levels of this biomarker (Figure 3(b)). Similarly, LDH levels increased with exposure time in all experimental conditions, principally in rats coexposed to As + PM_2.5_, although not statistically significant (Figure 3(c)).

**Figure 4:**
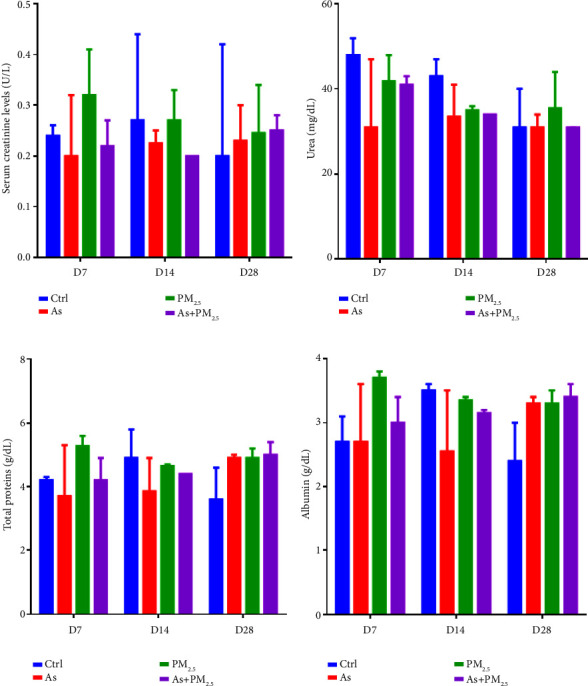
Biochemical test for kidney function. The serum level of creatine (a), urea (b), total proteins (c), and albumin (d) was assessed after 7, 14, and 28 days in the serum of rats exposed to As, PM_2.5_, or As + PM_2.5_ exposure to determine the kidney function, *n* = 4 per group. Data are presented as mean and SD. *p* = ns.

To analyze kidney function, we assessed creatine (Figure 4(a)), urea (Figure 4(b)), total proteins (Figure 4(c)), and albumin levels (Figure 4(d)) in the serum of rats exposed to As, PM_2.5_, and As + PM_2.5_. After analyzing these biomarkers, we did not observe significant changes in any of the levels of the analytes determined.

### 3.4. Alterations in Antioxidant Mechanisms

**Figure 5:**
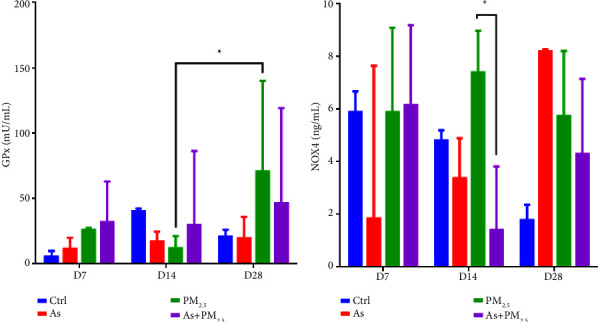
Alterations in antioxidant mechanisms. The antioxidant activity of the enzymes glutathione peroxidase (GPx) (a) and NADPH oxidase (NOX4) (b) was measured in the serum of rats exposed for 7, 14, and 21 days to As or PM_2.5_ or As + PM_2.5_. *n* = 4 per group. Data are presented as median (range). ^*∗*^*p*=0.04, PM_2.5_ (D14) vs PM_2.5_ (D28) to GPx and NOX4, respectively.

We assessed the activity of glutathione peroxidase (GPx) and NADPH oxidase (NOX4) in serum from exposed and unexposed rats to evaluate antioxidant mechanisms. The activity of GPx significantly decreased in the group exposed to PM_2.5_ on day 14 compared to day 28 (*p*=0.03) (Figure 5(a)). Interestingly, the activity of NOX4 increased on the same days of exposure. However, its activity decreased significantly in the presence of As + PM_2.5_ (*p*=0.04) (Figure 5(b)).

### 3.5. Differentially Expressed Gene (DEG) Analysis

Through the GEO platform, we analyzed gene expression from the data collected from similar exposure models to these pollutants. Induction and suppression of genes from the expression profiling assay of As exposure in a murine model (dataset from GSE19662) showed that the main cluster of genes altered by As exposure are those involved in oxidative stress (19 genes), cellular response to external stimuli (14 genes), cellular response to chemical stimuli (10 genes), and inflammatory response (6 genes) (Table 3).

Using the same platform, we analyzed data from an *in vitro* murine model of exposure to PM_2.5_ (dataset from GSE178513). After gene ontology analysis, we observed that the main gen clusters altered by PM_2.5_ exposure were related to oxidative stress (7 genes) and positive regulation of the apoptotic processes (2 genes) (Table 4).

### 3.6. Gene Expression Interaction Networks

**Figure 6:**
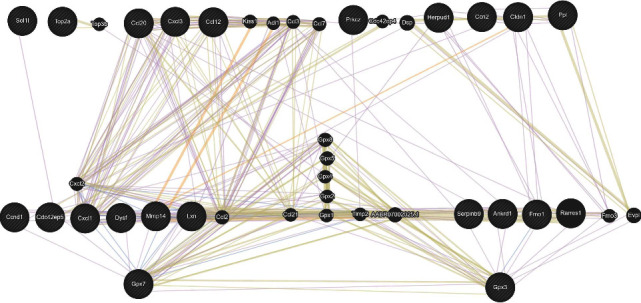
The protein-protein interaction of the DEGs with Gpx7 and Gpx3. The GeneMANIA platform was used for the interaction analysis. Two interactive clusters are presented, one with Gpx7 and one with Gpx3. In addition, an interactive cluster connects both Gpx7 and Gpx3. The yellow lines indicate a predicted protein-protein interaction, while the grey lines indicate that this interaction, or the relationship between the two proteins, occurs in the same pathway. In terms of size, this means there will be a greater probability of interaction.

We performed an interaction network map to correlate the biological functions of the upregulated genes with oxidative stress. As a first step, we analyzed the interactions in the mice exposed to As. In Figure 6, we observed two isoforms of glutathione peroxidase (GPx), GPx3 and GPx7, interacting with matrix metalloproteinase 14 (MMP14), chemokine chemoattractant 1 (CXCL1) and chemokine chemoattractant 21 (CCL21), tissue inhibitor of metalloproteinases 2 (TIMP2), serpin family B member 9 (SERPINB9), and ankyrin repeat domain 1 (ANKRD1), which have been implicated in tumor progression.

**Figure 7:**
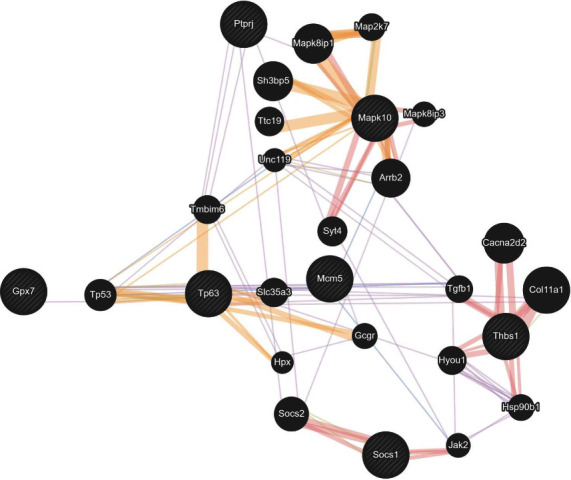
PM_2.5_ DEGs interact with Gpx7. A protein-protein interaction was performed to identify the protein-protein interaction between Gpx7 and the identified DEGs. The yellow lines indicate the predicted interaction between the molecules. The grey lines indicate that the molecules belong to the same pathway, and the orange lines indicate the physical interaction between the molecules. In terms of the size of the circle, the larger the circle, the more likely it is to be an interaction with the bound molecules.

We also analyzed the interactions caused by PM2.5 exposure, highlighting the interaction of GPx7 (Figure 7) with tumor protein 53 (TP53), which has been identified as a tumor suppressor protein.

## 4. Discussion

Environmental pollution is a worldwide public health problem that has increased during the last decades and is closely related to demographic boost and rapid industrialization. Among the vast environmental pollutants, arsenic (As) and particulate matter (PM) are two contaminants of interest for human health. Exposure to each of these pollutants has been linked to premature deaths as well as the development of different diseases [23–26]. For example, skin disorders, *diabetes mellitus* [27], cardiovascular diseases [4], and different types of cancer [28] are also observed in the population exposed to high levels of PM_2.5_ [29–31]. However, even though real-world exposures include simultaneous exposures to more than one pollutant, an experimental model has yet to be developed to explore the health effects of coexposures. Here, we proposed a novel method of coexposure to daily As in drinking water and to urban PM_2.5_ intratracheal instillation every other day.

This model's development helped us identify histological alterations in the lungs and hearts of animals exposed only to As and PM_2.5_ and in those coexposed to As and PM_2.5_. The PM concentration used in this model was determined through previous analysis [15]. In summary, we based our calculations on the approximate average concentration of PM in Mexico City, which is approximately 120 *µ*g/m^3^. Considering the typical person's daily inhalation of around 80 *μ*g of PM per mL of lining fluid, their average air intake, PM deposition rate, and lung volume, we determined that 64 *μ*g of PM would represent the concentration of PM that an average person would inhale on a typical day in Mexico City. Although this concentration should represent a daily exposure to PM, we decided to perform intratracheal exposure every other day to reduce the impact of stress that might cause manipulation in rats.

We observed cartilaginous metaplasia in the hearts of exposed animals to any of the pollutants alone or in combination. Previous reports have considered cartilaginous metaplasia a pathological structural condition related to aging, deficient mechanical activity, chronic diseases, and inflammatory processes [32, 33]. Although there is no direct evidence that exposure to arsenic or PM induces cartilaginous metaplasia, antecedents in rat models suggest the involvement of cellular and inflammatory molecules in the cartilaginous metaplasia [33]. Inflammation can damage tissue and stimulate the production of specific signaling molecules that attract and activate mesenchymal cells. These cells then differentiate into chondrocytes and form cartilage. Thus, high levels of inflammation and oxidative stress induced by As and PM_2.5_ [34–36] might be related to the development of cardiac metaplasia. The three exposed groups (As, PM_2.5_, and As + PM_2.5_) showed cartilaginous metaplasia; although time-related cartilaginous metaplasia areas did not show similarities among groups. An increment of this heart alteration may cause sudden cardiac death [37].

To corroborate the histological alterations of the heart, we determined lactate dehydrogenase (LDH) levels in serum from exposed animals. LDH is an enzyme that is involved in the production of energy in cells. While it is primarily associated with glycolysis, it has also been implicated in other cellular processes, such as regulating gene expression and maintaining cellular homeostasis. There is limited information available regarding the relationship between LDH and cardiac cartilaginous metaplasia. However, studies have suggested that LDH may play a role in the pathogenesis of other cardiac diseases, such as heart failure and ischemic heart disease [38]. Elevated levels of LDH have been associated with poor prognosis in these conditions, and it has been proposed that LDH may contribute to myocardial injury and inflammation. Further research is needed to determine the relationship between LDH and cardiac cartilaginous metaplasia. Similarly, creatine kinase-MB (CK-MB) is another cardiac enzyme that is released into the bloodstream when there is damage to the heart muscle. Studies have suggested that CK-MB may play a role in developing other cardiac diseases, such as heart failure and ischemic heart disease [39]. These findings coincide with previous studies that found high levels of LDH and CK-MB after acute As exposure [40]. Interestingly our results also showed an effect produced by the exposure to PM_2.5_, which was additive in coexposed groups.

On the other hand, the three experimental groups showed a time-dependent increasing inflammatory trend, getting the highest levels of lung inflammation after 28 days of exposure. Interestingly, the lung inflammation showed an additive effect when rats were exposed simultaneously to As and PM_2.5_. Different studies have reported lung inflammation in animals exposed to As [41, 42] or PM_2.5_ [43, 44]. However, this is the first report that shows the additive inflammatory effect caused by simultaneous exposure to As and PM_2.5_. High levels of inflammation might be linked to the development of cartilaginous metaplasia in the heart.

Both As and PM_2.5_ exposure have been linked to oxidative stress and inflammation [45, 46], which can lead to various adverse health effects. Glutathione peroxidase (GPx) is an antioxidant enzyme that helps to protect cells from oxidative damage by scavenging harmful reactive oxygen species (ROS). GPx activity is also commonly used to measure oxidative stress indirectly, and increased GPx activity may suggest an upregulation of the antioxidant system to combat increased ROS production [47]. Consistently with the antecedents, we found a time-dependent increase in the GPx activity in animals exposed to PM_2.5_ on day 28 compared to day 14. The difference observed may be related to the content of polycyclic aromatic hydrocarbons (PAHs) in the PM_2.5_ used to perform these experiments. This is because PAHs can undergo metabolic activation to form reactive intermediates that can generate ROS [48]. Interestingly, following 14 days of exposure to As + PM_2.5_, a decrease in NOX4 levels was observed in the As + PM_2.5_ group. The decrease in NOX4, a known contributor to cellular oxidative stress, may be associated with the induction of oxidative stress and inflammation by both As and PM_2.5_. Our initial interpretation proposes that the suppression of NOX4 levels could be a result of a modulation of inflammatory signals as a response to the combined exposure. Nevertheless, further experiments are needed to have a better understanding of the observations.

Using the GEO platform, we analyzed the differential gene expression reported in diverse exposure models to As or PM_2.5_. Similar to our results, the gene expression of the studied models showed overexpression of antioxidant mechanisms, such as GPx. By following up with bioinformatical analysis, the mapping showed an interaction between GPx and other genes related to the control/progression of cancer, such as MMP14 [49], TIMP2 [50], SERPINB9 [51], ANKRD1 [52], TP53 [53], and BTG2 [54]. Besides, we observed that GPx3 and GPx7 were the two isoforms expressed after As or PM_2.5_ exposure. Studies have suggested that GPx3 and GPx7 protect the lungs from oxidative stress and inflammation. Decreased expression or activity of these enzymes has been linked to lung injury and inflammation in various animal models [55]. Besides, GPx7 showed increased expression in multiple types of tumors [56]. Furthermore, clinical studies have shown that decreased levels of GPx3 in the lungs are associated with an increased risk of acute lung injury in critically ill patients. These findings highlight the importance of GPx3 and GPx7 in maintaining lung homeostasis and protecting against inflammation and injury.

These data suggest that continuous coexposure to arsenic and PM_2.5_ can additively affect inflammation, ROS production, and GPx levels in the body. Furthermore, coexposure to arsenic and PM_2.5_ can lead to a more significant increase in oxidative stress and inflammation than exposure to either pollutant alone. This may be because both pollutants can activate similar inflammatory pathways and increase ROS production, leading to a cascade of harmful effects in the body. Continuous coexposure to arsenic and PM_2.5_ can significantly impact the body's antioxidant defense system, leading to chronic inflammation and oxidative stress. This can increase the risk of various health problems, including cardiovascular disease, respiratory disease, and cancer.

Our study showed that coexposure to As and PM_2.5_ in rats induced cartilaginous metaplasia in the heart and increased lung inflammation. These effects were additive in the coexposed group, suggesting that simultaneous exposure to these pollutants may exacerbate their adverse health effects. Our findings also showed increased LDH and CK-MB levels, which may indicate a cardiac injury and inflammation. In addition, we observed a time-dependent increase in GPx activity in animals exposed to PM_2.5_ and As + PM_2.5_, suggesting an upregulation of the antioxidant system to combat increased ROS production. Overall, our results highlight the importance of studying the health effects of coexposure to environmental pollutants, as individuals are often exposed to multiple contaminants simultaneously in the real world. Further research is needed to elucidate the underlying mechanisms by which As and PM_2.5_ induce cartilaginous metaplasia and to explore potential therapeutic interventions to mitigate the adverse health effects of these pollutants.

## 5. Conclusions

1. Simultaneous exposure to PM_2.5_ and arsenic synergistically induces high levels of lung inflammation.
2. Heart cartilaginous metaplasia was observed in animal groups exposed to arsenic, PM_2.5_, or arsenic + PM_2.5_. These alterations appeared after seven days of exposure.
3. Biochemical markers for cardiac function also showed alterations after exposure to arsenic, PM_2.5_, or arsenic + PM_2.5_, which might corroborate heart alterations.
4. Exposure to arsenic, PM_2.5_, or arsenic + PM_2.5_ did not cause kidney alterations.
5. NOX4 activity decreased significantly in the presence of arsenic + PM_2.5_.

## Figures and Tables

**Table 1:** Elemental composition of PM_2.5_ sampled in the urban zone of Mexico City.

Elemental composition	PM_2.5_	PM_2.5_
	*μ*g/mg	%
Na	0.37	0.037
Mg	0.08	0.008
Al	1.15	0.115
Si	2.75	0.275
P	0.22	0.022
S	4.31	0.431
K	2.07	0.207
Ca	3.18	0.318
Ti	1.36	0.163
V	0.19	0.019
Cr	0.01	0.001
Mn	0.05	0.005
Fe	2.63	0.263
Ni	0.10	0.01
Cu	0.22	0.022
Zn	0.27	0.027
Se	0.02	0.002
Br^−^	0.03	0.003
Sr	0.01	0.001
Pb	0.71	0.071

**Table 2:** Polycyclic aromatic hydrocarbons (PAHs) content in PM_2.5_ sampled in the urban zone of Mexico City.

Hydrocarbon composition	PM_2.5_	PM_2.5_
	*μ*g/mg	%
Naphthalene	5.58	0.55
Fluorene	11.67	1.16
Phenanthrene	29.37	2.93
Fluoranthene	42.83	4.28
Pyrene	54.94	5.4
Retene	6.74	0.67
Benz[a]anthracene	27.90	2.79
Cyclopenta[cd]pyrene	0.00	0
Chrysene	54.36	5.43
Benzo[b]fluoranthene	92.62	9.26
Benzo[k]fluoranthene	51.38	5.13
Benzo[j]fluoranthene	36.18	3.61
Benzo[e]pyrene	67.10	6.71
Benzo[a]pyrene	78.13	7.81
Perylene	18.41	1.84
Dibenz[a,h]anthracene	3.76	0.37
Indeno[1,2,3-c,d]pyrene	103.96	10.39
Benzo[ghi]perylene	138.54	13.85

**Table 3:** Biological processes of a subgroup of 49 genes upregulated by arsenic exposure through a gene ontology (GO) analysis.

Biological process	Number of genes involved	*P* value
Stress response	19	8.06*E* − 16
Cellular response to chemical stimulus	14	3.43*E* − 09
Cellular response to organic substance	10	6.07*E* − 06
Inflammatory response	6	2.72*E* − 06

**Table 4:** Biological processes of a subgroup of 9 genes upregulated by PM_2.5_ exposure through a gene ontology (GO) analysis.

Biological process	Number of genes involved	*P* value
Stress response	7	2.72*E* − 06
Positive regulation of the apoptotic process	2	4.05*E* − 06

## Data Availability

All technical and scientific data used to support the findings of this study are included within the article. Nevertheless, the authors are willing to contribute to researchers who would like to reproduce their different tests.
